# Treatment with P28GST, a schistosome-derived enzyme, after acute colitis induction in mice: Decrease of intestinal inflammation associated with a down regulation of Th1/Th17 responses

**DOI:** 10.1371/journal.pone.0209681

**Published:** 2018-12-28

**Authors:** Aurore Sarazin, Arnaud Dendooven, Marie Delbeke, Solène Gatault, Aurélien Pagny, Annie Standaert, Christel Rousseaux, Pierre Desreumaux, Laurent Dubuquoy, Monique Capron

**Affiliations:** 1 Univ. Lille, Inserm, CHU Lille, U995 – LIRIC – Lille Inflammation Research International Center, Lille, France; 2 IBD Biotech, Lille, France; Future University, EGYPT

## Abstract

**Background:**

P28GST, a 28Kd glutathione *S*-transferase enzymatic protein derived from a schistosome helminth prevents experimental colitis when administered subcutaneously in the presence of adjuvant by decreasing pro-inflammatory Th1/Th17 response. Given the antioxidant properties of P28GST, we evaluated its anti-inflammatory potential when administered locally after colitis induction in the absence of adjuvant.

**Methods:**

Colitis was induced in BALB/c mice by rectal administration of TNBS, followed by two intraperitoneal injections of P28GST at day 1 and day 2. Mice were sacrificed 48h after TNBS administration and evaluated for macroscopic and histological scores, myeloperoxidase (MPO) quantification and cytokine messenger RNA expression in the colonic tissues.

**Results:**

Both clinical and histological scores significantly decreased in mice treated with P28GST at 5 or 50μg/kg when compared to vehicle- treated mice. A significant reduction of MPO was detected in colonic tissues from P28GST–treated mice, similarly to mice treated with methylprednisolone as the reference treatment. Pro-inflammatory cytokines TNF, IL-1β, and IL-6, mRNA as well as serum levels were down-regulated in mice colonic tissues treated with P28GST at 5 or 50μg/kg. In addition, a significant decrease of mRNA expression levels of T-bet, and ROR-γ, respective markers of Th1 and Th17 cells was observed. Whereas no significant effect was detected on Gata3 mRNA, a marker of Th2 cells, the Arg/iNOS mRNA levels significantly increased in P28GST-treated mice, suggesting the induction of M2 macrophages.

**Conclusions:**

These findings provide evidence that P28GST injected locally after colitis induction induces a potent decrease of colitis inflammation in mice, associated to downregulation of Th1/Th17 response, and induction of anti-inflammatory alternatively activated macrophages.

## Introduction

Inflammatory Bowel Diseases (IBD) are immune-mediated inflammatory disorders of the digestive tract including Crohn’s disease (CD) and ulcerative colitis (UC). The pathogenesis of IBD is not completely understood but it is generally admitted that it is caused by inappropriate activation of the mucosal immune system, which results in a state of chronic inflammation associated with dysregulation of the cytokine network. Indeed, CD is associated with a T helper type 1 (Th1)/Th17 response, with pro-inflammatory Th1 cells expressing large amounts of pro-inflammatory cytokines such as Interferon-γ (IFN-γ), Tumor Necrosis Factor (TNF) and Interleukin-1β (IL-1β) in the intestinal mucosa. Th17 cells massively infiltrate the inflamed intestine of patients where they produce IL-17 that mediates pro-inflammatory functions including upregulation of TNF and IL-1β, recruitment of neutrophils and secretion of matrix metalloproteinases by intestinal fibroblasts [1].

There is currently no cure for CD and most strategies consist in reducing the intensity of chronic inflammation and shortening the episodes of acute inflammation. Controlling the expression and the activity of pro-inflammatory cytokines is an approach that needs to combine anti-inflammatory efficacy with low suppressive effects on immune defense. A number of treatment options are available including several TNF-alpha inhibitors (Infliximab, Adalimumab). Unfortunately, many patients fail to respond to these therapies or develop severe side effects [2,3]. Other approaches consist in inhibiting the migration of lymphocytes into the inflamed tissue with the use of selective anti-adhesion molecules (Vedolizumab, Natalizumab) [4]. Alternatively, antioxidant defense is an interesting therapeutic strategy as oxidative stress could be a major contributing factor to the tissue injury that characterizes CD [5].

The use of helminth properties is an emerging and promising strategy to modulate immune disorders. Helminths are worm parasites that can establish in human gastrointestinal tract and modulate host immune defenses; they may even block the inflammatory pathways that are implied in autoimmune diseases. In developing countries, high incidence of parasitic infections has been proposed to be correlated with a significantly low incidence of autoimmune diseases, whereas intensive “deworming” strategies might have disturbed long-term developed regulatory networks, and may be, at least partly, responsible for the emergence of inflammatory disorders [6,7].

Following the early migration phase, immune response to helminths is generally polarized at the establishment phase towards a Th2 response, preventing Th1 or Th17 immune response, and associated with increased Th2 cytokines. Helminth products can cause the differentiation of macrophages toward the M2 phenotype, and promote the production of immunoregulatory molecules such as IL-10 and TGF-β [7]. These remarkable properties of helminths to shift immune response from pro-inflammatory Th1 to anti-inflammatory Th2/Tregs has led to the concept of helminth therapy, based on the use of helminths or preferably helminth products to treat auto immune disorders [7].

Helminths have been used in experimental models to improve intestinal inflammation, notably in reducing severity scores, decreasing histological inflammation and downregulating inflammatory cytokine production. The immunomodulatory properties of schistosomes have been validated in some models of immunopathology. The effects of excretion-secretion products by eggs or adult worms in the regulation of experimental colitis have also been described. Omega-1 is a glycoprotein secreted by schistosome eggs that has been shown to drive Th2 polarization [8]. Among helminth secreted products, only a few molecules or proteins have been fully characterized. P28GST [9], a major enzymatic protein identified in schistosomes and developed as a vaccine candidate against schistosomiasis [10–12] has been recently proposed as a potent immunoregulatory molecule able to control intestinal inflammation [13].

Our previous work which showed that schistosome P28GST induced a strong mucosal immune response associated with a Th2 response and production of IL-10 led us to evaluate its immunomodulatory properties in experimental colitis. We could show that systemic immunization of rats and Balb/c mice with recombinant *S*. *haematobium* (rSh) P28GST in the presence of adjuvant ameliorates intestinal inflammation in a preventive mode through modulation of Th1 response [13]. P28GST which successfully passed phase 1 clinical trials for safety and immunogenicity studies (NCT01512277) is currently under investigation in CD patients in a phase 2 clinical trial (NCT02281916).

Since P28GST can express both immunomodulatory and anti-oxidant properties, we can assume that in addition to its role in prevention of mucosal inflammation, needing a pro-Th2 adjuvant, it can also be effective in reducing inflammation in a curative mode. The aim of the present study was to evaluate the effect of a local treatment with P28GST on gut inflammation when administered after TNBS-colitis induction in mice.

## Materials and methods

### Animals and ethical considerations

Housing and all of experimental procedures were approved by the local Animal Care Ethical Committee (Comité d’Ethique en Expérimentation Animale Nord—Pas de Calais) (Approval number. 352012). Experimental procedures were performed in accordance with French law and national guideline for the Care and Use of Laboratory Animals and the guidelines of the European Union. Animals were housed under specific pathogen-free conditions in animal holding facilities in Institut Pasteur de Lille (UE number: B59-350009). Specific methods (acclimation period, gentle handling, etc.) as well as appropriate medications to prevent or eliminate stress and pain (ketamin, xylazin, etc.) and euthanasia (pentobarbital) were used. Eight weeks old female BALB/c mice of approximately 20g in weight were purchased from Janvier Labs (Le Genest Saint-Isle, France).

### Chemicals and reagents

Recombinant Sh P28GST protein was expressed in *Saccharomyces cerevisiae* culture and purified under Good Manufacturing Practice conditions by Eurogentec S.A (Seraing, Belgium). Batches of P28GST (batchM-BIX-P03-225a) were conserved lyophilized in NH_4_HCO_3_ 10mM and 2.8% lactose. Control batches contained only NH_4_HCO_3_ and lactose. These preparations were re-suspended extemporaneously using NaCl 0.9% at the appropriate concentrations. Lyophilized GST from human placenta was purchased from Sigma-Aldrich and prepared according to the manufacturer’s instructions. For heat-inactivation, P28GST and GST from human placenta were incubated 15 minutes at 95°C. Quercetin was used as positive control (Sigma-Aldrich, Saint-Louis, Missouri). A commercial preparation of Methylprednisolone (Mylan, Canonsburg, Pennsylvania) was resuspended extemporaneously using 0.9% NaCl solution and administrated to mice by intraperitoneal injections at (2 mg/kg) at day 1 and day 2 after colitis induction. 2,4,6- Trinitrobenzenesulfonic acid (TNBS) (Sigma-Aldrich, Saint-Louis, Missouri) was used for colitis induction.

### Induction of colitis

A standardized murine TNBS-colitis model was used. Five groups of 12–15 mice were randomly constituted. Briefly, under anesthesia with Xylazine (SEDAXYLAN 20 mg/ml, EUROVET Animal Health BV, Netherlands, 50 mg/kg) and Ketamine (Ketamine 1000, Virbac, France, 50 mg/kg), 40 μL of 150 mg/kg TNBS solution in 50% ethanol was slowly administered in the colon via a catheter (internal diameter 0.3mm; external diameter 0.7mm; Folioplast, Sarcelles, France) until the tip was 4 cm proximal to the anus [14]. Control mice received only 50% ethanol.

Mice were then treated with 2 intraperitoneal (ip) injections of either vehicle or 5, 50, 500 μg/kg of P28GST or methylprednisolone at 2mg/kg as reference treatment. Injections of P28GST were administrated at day 1 and day 2 after TNBS administration. Mice were weighed daily and controlled for clinical and behavioral signs of pain. Body weight of mice was recorded before diet and on sacrifice day. Results are expressed in % of loss of initial weight. They were sacrificed 48h after TNBS administration. Colons were removed and opened longitudinally. Stools were discarded and an examination of the whole colon was performed to evaluate the macroscopic lesions according to Wallace scoring method. Then a colon specimen located at 1 cm of the distal part of the colonic segment was fixed in 4% paraformaldehyde acid and embedded in paraffin for histological analysis. The next 1 cm segment was immediately frozen, stored at -80°C and used later for quantification of myeloperoxidase (MPO) and RNA extractions.

### Macroscopic and histological analysis

Macroscopically visible damage of the opened colonic segment was blindly scored on a 0–10 scale using the Wallace scoring method based on criteria reflecting inflammation, thickening of the bowel and the extent of ulceration [15].

Morphometric analysis was performed on May Grunwald-Giemsa stained 4μm transverse sections from colon samples fixed at 4°C overnight in 4% (v/v) formaldehyde and embedded in paraffin. The extent of colonic inflammatory damages was assessed blindly according to Ameho criteria [16]. This grading on a scale from 0 to 6 takes into account the degree of the inflammatory infiltrate and the presence of ulceration or necrosis.

### Quantification of tissue myeloperoxidase and blood cytokines by ELISA

For myeloperoxidase assay, each tissue sample was homogenized in a buffer containing 200mM NaCl, 10mM Tris Base, 5mM EDTA and 1mM PMSF with an Ultra Turax T10 (IKA, Staufen, Germany). The homogenates were then centrifuged at 1,500g for 15 minutes at 4°C to pellet the insoluble debris. Total protein concentration was measured in the supernatants with the Bradford method [17] using the Quick Start Bradford Protein Assay (Bio-Rad, Hercules, CA). Myeloperoxidase (MPO) levels from colonic specimens were then quantified using the mouse MPO ELISA kit from Hycult Biotech (Uden, Netherlands) according to the manufacturer’s instructions [18]. Results are expressed as MPO (ng)/ total protein (mg) ratios.

For quantification of blood cytokines, blood samples were obtained by cardiac puncture of anesthetized mice just before sacrifice. Serum samples were collected after centrifugation and were stored at -80°C. Cytokine levels in sera were measured by Luminex assay as recommended by the manufacturer (R&D Systems, Minneapolis, Minnesota). Multiple analytes can be detected in the same sample with this method. Briefly, color-coded magnetic microbeads coated with analyte-specific antibodies were incubated with the samples. Captured analytes were detected on a dual-laser flow-based detection instrument (Bio-Plex 200 System, Bio-Rad, Hercules, CA) using a cocktail of biotinylated detection antibodies and a streptavidin-phycoerythrin conjugate.

### RNA extraction and analysis of mRNA expression by quantitative PCR

Mice colon samples were homogenized with an Ultra Turax T10 (IKA, Staufen, Germany) and RNA was isolated with NucleoSpin RNA II kit (Macherey-Nagel, Hoerdt, France) according to the manufacturer’s protocol. The RNA concentration and purity was determined by absorbance at 260nm and 280nm on a NanoDrop 1000 (Thermo Fisher Scientific, Waltham, Massachusetts). One μg of RNA was used to synthesize cDNA using a High Capacity cDNA Reverse Transcription Kit (Applied Biosystems, Foster City, California). Real-time quantitative PCR (q-PCR) was carried out with the Fast Power SYBR Green PCR master mix (Applied Biosystems, Foster City, California) according to the manufacturer’s instructions. The analyses were performed with an ABI StepOnePlus (Applied Biosystems, Foster City, California). Glyceraldehyde-3 phosphate dehydrogenase (GAPDH) was used as housekeeping gene. The Primer sequences are listed in Table 1.

**Table 1:** Primer sequences for qPCR.

Gene	Primer	Sequence
**GAPDH**	Forward	ATGGGAAGCTTGTCATCAACG
	Reverse	GGCAGTGATGGCATGGACTG
**TNF**	Forward	CATCAGTTCTATGGCCCAGACCCT
	Reverse	GCTCCTCCACTTGTTGTTTTGCTA
**IL-1β**	Forward	AGCTCTCCACCTCAATGGAC
	Reverse	AGGCAACAGGTATTTTGTCG
**IL-6**	Forward	GTTCTCTGGGAAATCGTGGA
	Reverse	CAGAATGCCATTGCACAAC
**Arg**	Forward	CAGAAGAATGGAAGAGTCAG
	Reverse	CAGATATGCAGGGAGTCACC
**iNOS**	Forward	CAGCTGGGCTGTACAAACCTT
	Reverse	CATTGGAAGTGAAGCGTTTCA
**Tbet**	Forward	GCCAGGGAACCGCTTATATG
	Reverse	GACGATCATCTGGGTCACATTGT
**GATA3**	Forward	CATTACCACCTATCCGCCCTATG
	Reverse	CACACACTCCCTGCCTTCTGT
**RORɤ**	Forward	GAAAGCAGGAGCAATGGAAG
	Reverse	GATGGAAAGCCAGTTCCAAA
**COX2**	Forward	GCTGTACAAGCAGTGGCAAA
	Reverse	GCTCGGCTTCCAGTATTGAG

Abbreviations: GAPDH, glyceraldehyde-3 phosphate dehydrogenase; TNF, tumor necrosis factor; IL-1β, interleukin-1β; IL-6, interleukin-6; COX2, Cyclo-oxygenase-2; Arg1, Arginase-1; iNOS, inducible NO-synthase; Tbet, T-box 21; GATA3, GATA binding protein 3; RORɣ, RAR related orphan receptor C.

### Determination of GST enzymatic activity

Enzymatic activity of 20μL solution of P28GST was tested in the presence of 1mM glutathione (Sigma-Aldrich, Saint-Louis, Missouri) in 50mM sodium phosphate buffer (pH 6.5). The reaction was started by the addition of CDNB (1-chloro-2,4-di-nitrobenzene) (Sigma-Aldrich, Saint-Louis, Missouri) to a final concentration of 1mM. The reaction was run at room temperature and absorbance at 340nm was measured every 15 seconds during 2 minutes [19]. GST specific activity (U/mg protein) was calculated using results of three independent experiments.

### Evaluation of antioxidant activity of P28GST

The radical scavenging activity of P28GST was tested spectrophotometrically by [2,2′-azino-bis (3-ethylbenzothiazoline-6-sulfonic acid)] ABTS^•^+ cation decolorization assay (Sigma-Aldrich, Saint-Louis, Missouri) [20]. 10 μL of sample (P28GST, Human placenta GST, Quercetin or vitamin E) (Sigma-Aldrich, Saint-Louis, Missouri) were mixed with 4 ml of ABTS radical solution and incubated 30 min in the dark. The decrease in absorbance at 730 nm was measured. The Scavenging activity (%) was calculated as follows: Scavenging activity (%) = (control Abs − sample Abs)/ (control Abs) × 100.

### Statistical analysis

Each animal experiment was performed four times, measures of GST enzymatic activity and scavenging activity were performed three times. The graphs and statistical analyses were generated using GraphPad Prism 5 (GraphPad Software, Inc., La Jolla, California). Statistical significance was calculated using one-way analysis of variance (ANOVA) followed by *post hoc* test. P < 0.05 was considered significant. All data are expressed as mean ± SEM. *, p< 0.05; **, p< 0.01, ***, p< 0.001 versus control groups.

## Results

### Treatment with P28GST after TNBS colitis induction reduced inflammation scores

**Fig 1:**
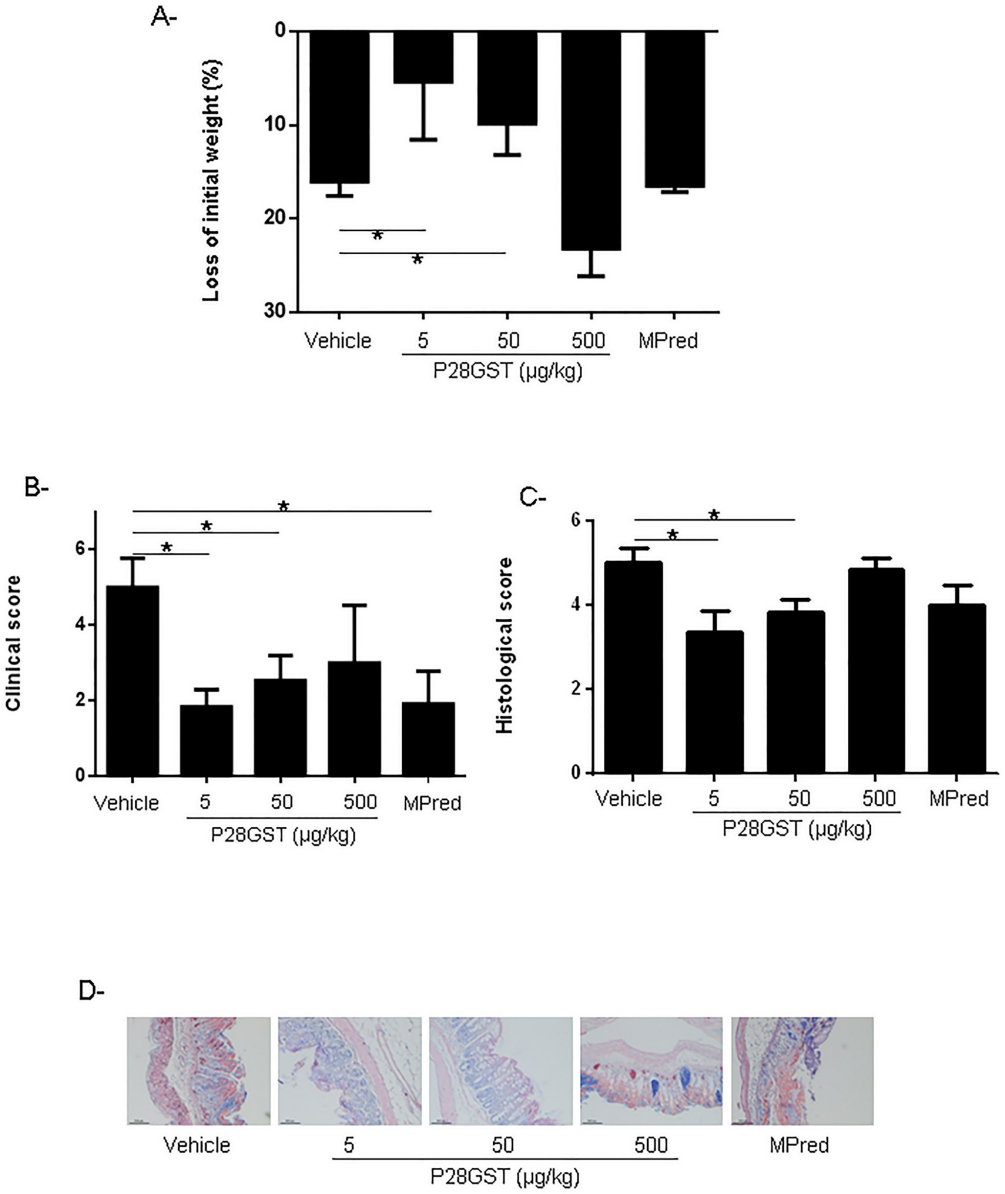
Treatment of Balb/c mice with P28GST after TNBS induction of colitis decreases intestinal inflammation. TNBS colitis was induced in all groups. Balb/c mice were treated with 2 ip injections of either P28GST (5, 50 and 500 μg/kg) or methylprednisolone (2mg/kg) at day 1 and day 2 after TNBS injection. All mice were sacrificed 2 days after TNBS injection. A, % of loss of initial weight. B, Clinical scores and C, Histological scores. D, May-Grunwald and Giemsa staining of paraffin-wax-embedded sections of mice colons (x10). Results are expressed as means ± SEM of four similar experiments; n = 10–12 mice per group. *p<0.05.

Dose–response experiments with P28GST and comparison with methylprednisolone as reference treatment were performed in this “curative” mode. As shown in Fig 1A, 1B and 1C, animals treated with 5 or 50 μg/kg P28GST after colitis induction exhibited significantly decreased loss weight as well as clinical and histological scores, when compared with vehicle treated positive controls. Intraperitoneal administration with the lowest doses of P28GST induced the same effects as the reference treatment with methylprednisolone, whereas the highest concentration of P28GST (500 μg/kg) had no effect on colitis. Compared with vehicle-treated mice, limited histological damage was observed in colons from mice treated with P28GST at 5 or 50 μg/kg (Fig 1D). The doses of 5 and 50 μg/kg were selected for further investigations. The loss of typical colonic crypt structure in control mice was accompanied by submucosal swelling, microclot formations, whereas a significant reduction of necrosis of the colonic mucosa as well as a significant recovery of colonic architecture was observed in P28GST-treated animals (Fig 1D).

### Decrease of inflammation markers by P28GST treatment

**Fig 2:**
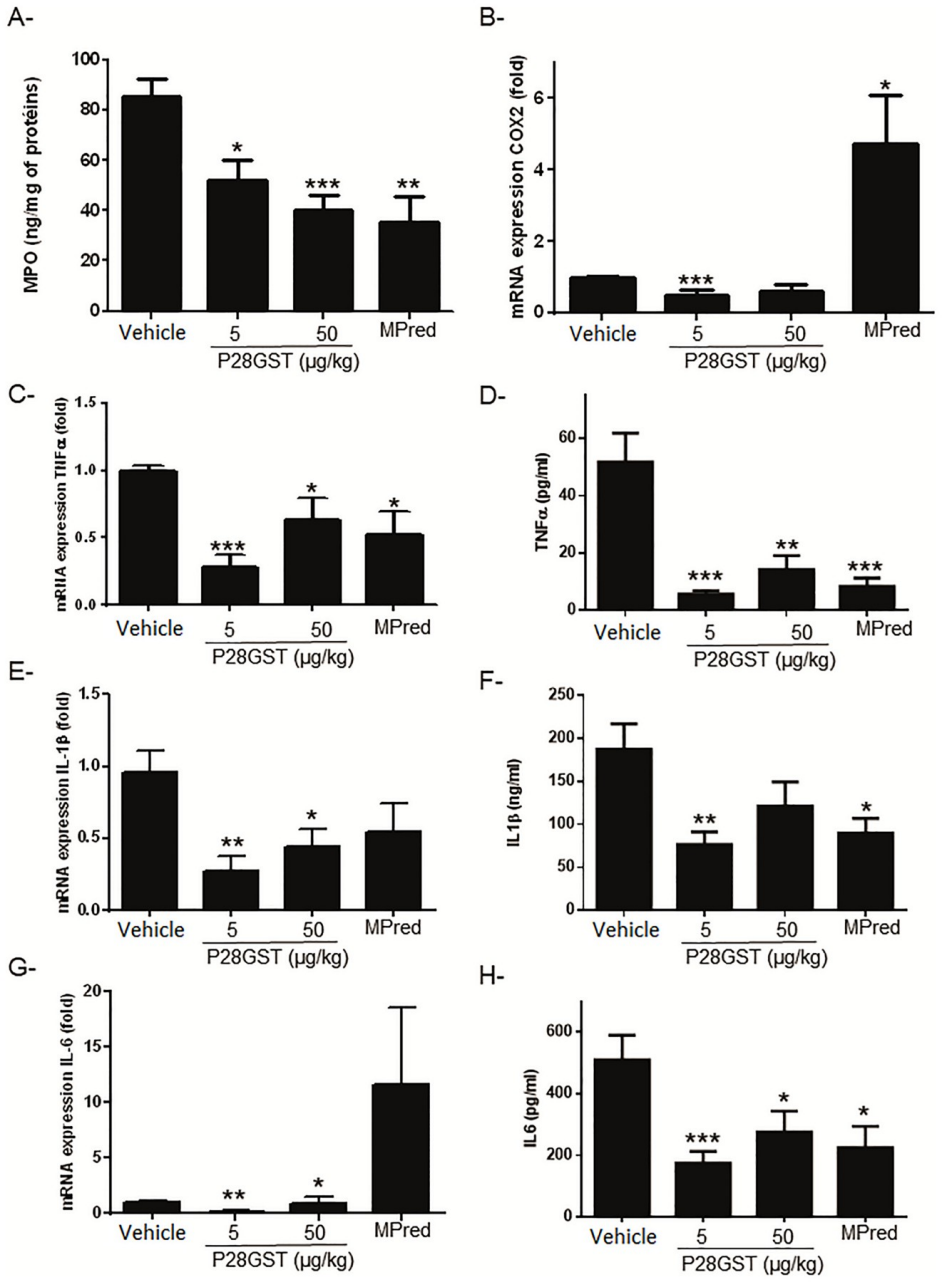
Inflammatory markers are decreased after treatment with P28GST. Mice were treated with 2 ip injections of either P28GST or methylprednisolone after TNBS-injection. Colons and peripheral blood were collected from each mouse 2 days after TNBS injection. A, MPO was quantified by ELISA in mice colons. MPO levels expressed as ng per mg of total proteins are lower in treated animals. B, Cox-2; C, Tumor necrosis factor (TNF); E, IL-1β and G, IL-6 mRNA expression was assessed by quantitative PCR in mice colons. D, F, H, Pro-inflammatory cytokines (TNF, IL-1β and IL-6) concentrations were investigated in each serum sample. Data are presented as mean value ± SEM of four similar experiments. N = 10–12 per group. *p<0.05; **p<0.01; ***p<0.001.

In the TNBS colitis model, colonic damage was associated with a high level of MPO (80 ng/mg of total proteins), a marker of neutrophil infiltration into the inflamed tissue. In mice treated with 5 or 50 μg/kg P28GST, a significant reduction of MPO was detected in mice colons, similarly to mice treated with methylprednisolone (Fig 2A). These results are in accordance with the scores and suggest a decreased neutrophil infiltration in the colonic tissues.

The mRNA encoding Cox-2, an inducible enzyme required for prostaglandins (PG) synthesis also significantly decreased in mice treated with the 5μg dose of P28GST, which is not the case for mice treated with methylprednisolone (Fig 2B).

This MPO decrease was accompanied with an important drop reaching more than 75% reduction in the colonic mRNA expression of the pro-inflammatory cytokines TNF, IL-1β, and IL-6, for the same doses of P28GST (5 or 50 μg/kg) (Fig 2C, 2E and 2G). Treatment with the lowest dose of P28GST induced lower levels of TNFα, IL-1β, and IL-6 mRNA than the reference treatment with methylprednisolone.

In parallel, the effect of P28GST was also investigated on the serum levels of pro-inflammatory cytokines: TNFα, IL-1β, and IL-6 (Fig 2D, 2F and 2H). A TNFα drastic diminution (>50%) was observed for the 2 doses of P28GST as well as after methylprednisolone treatment. Concerning IL-1β, a statistical decrease for P28GST 5μg/kg (p<0.01) and reference treatment (p<0.05) was demonstrated (Fig 2F). Similar results were observed for IL-6 (Fig 2H).

### Decrease of Th1/Th17 regulatory markers by P28GST treatment

**Fig 3:**
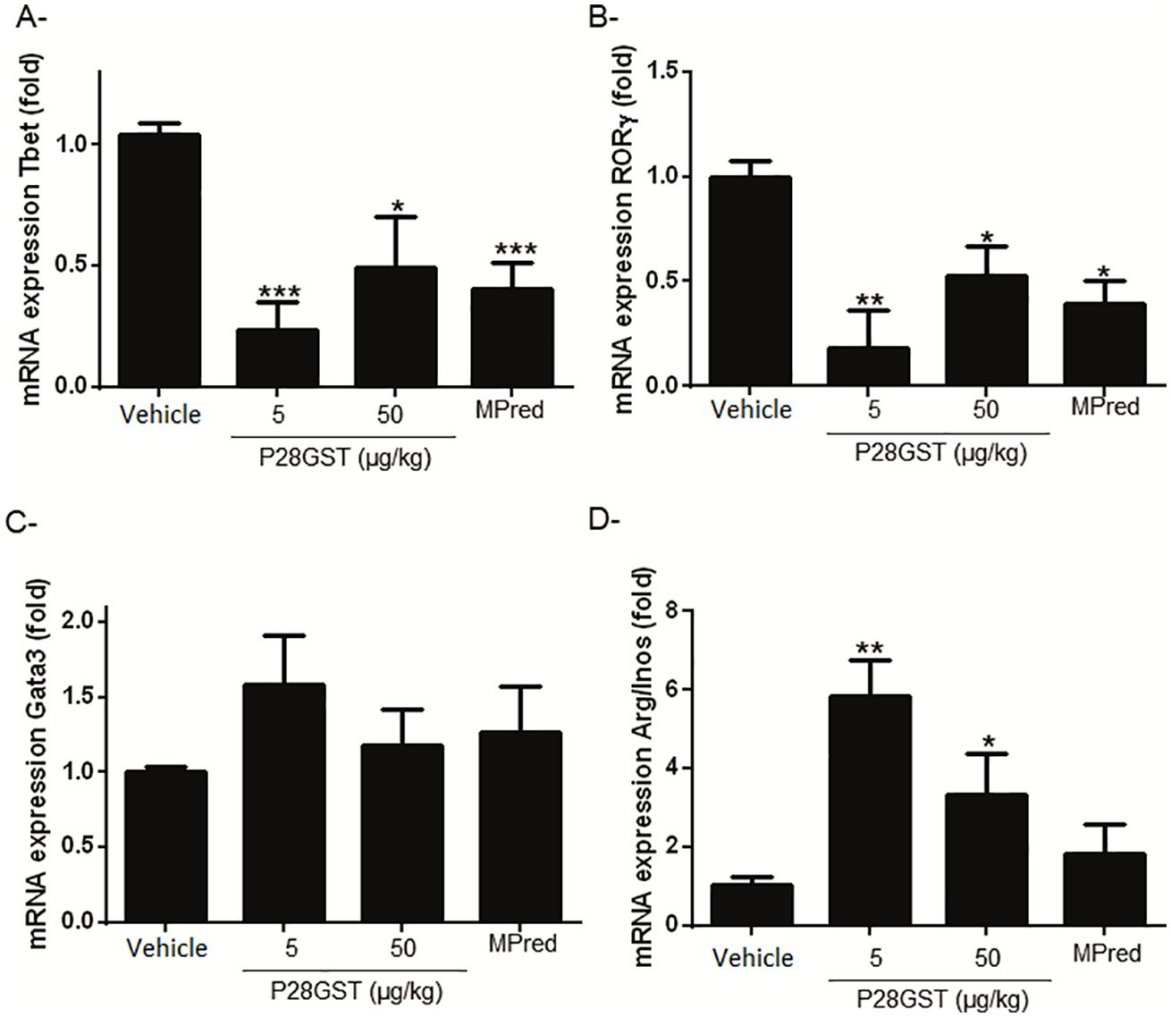
Decrease of Th1/Th17 markers after P28GST treatment and predominance of Alternatively Activated Macrophages. Mice were treated with 2 ip injections of either P28GST or methylprednisolone after TNBS injection. Colons were removed after sacrifice2 days after TNBS injection. Tbet (A), RORγ (B) and GATA3 (C) respectively markers of T helper (Th) 1, Th17 and Th2 cells, and Arg and iNOS (D), respectively markers of M2 and M1 macrophages, mRNA expression was assessed by quantitative PCR in mice colons. Results are expressed as means ± SEM from four similar experiments, n = 10–12 mice per group. *p<0.05; **p<0.01; ***p<0.001.

Treatment with P28GST after TNBS-colitis induction significantly reduced the proportion of Th1 cells in the colons evaluated by Tbet mRNA levels (Fig 3A), and decreased in a dose-dependent manner the RORγ mRNA levels, as marker of Th17 cells (Fig 3B). However, no significant effect on Gata3 mRNA, as marker of Th2 cells could be observed (Fig 3C).

As indicator of the ratio of M2/M1 macrophages, we evaluated the Arg/iNos mRNA ratio in the colons (Fig 3D). A significant increase of this ratio was observed in mice which have been treated with 5 or 50 μg/kg P28GST, suggesting a predominance of M2 macrophages in P28GST—treated mice, in contrast to treatment with methylprednisolone, which had no effect on this parameter.

### P28GST enzymatic activity is necessary to induce anti-inflammatory effects

**Fig 4:**
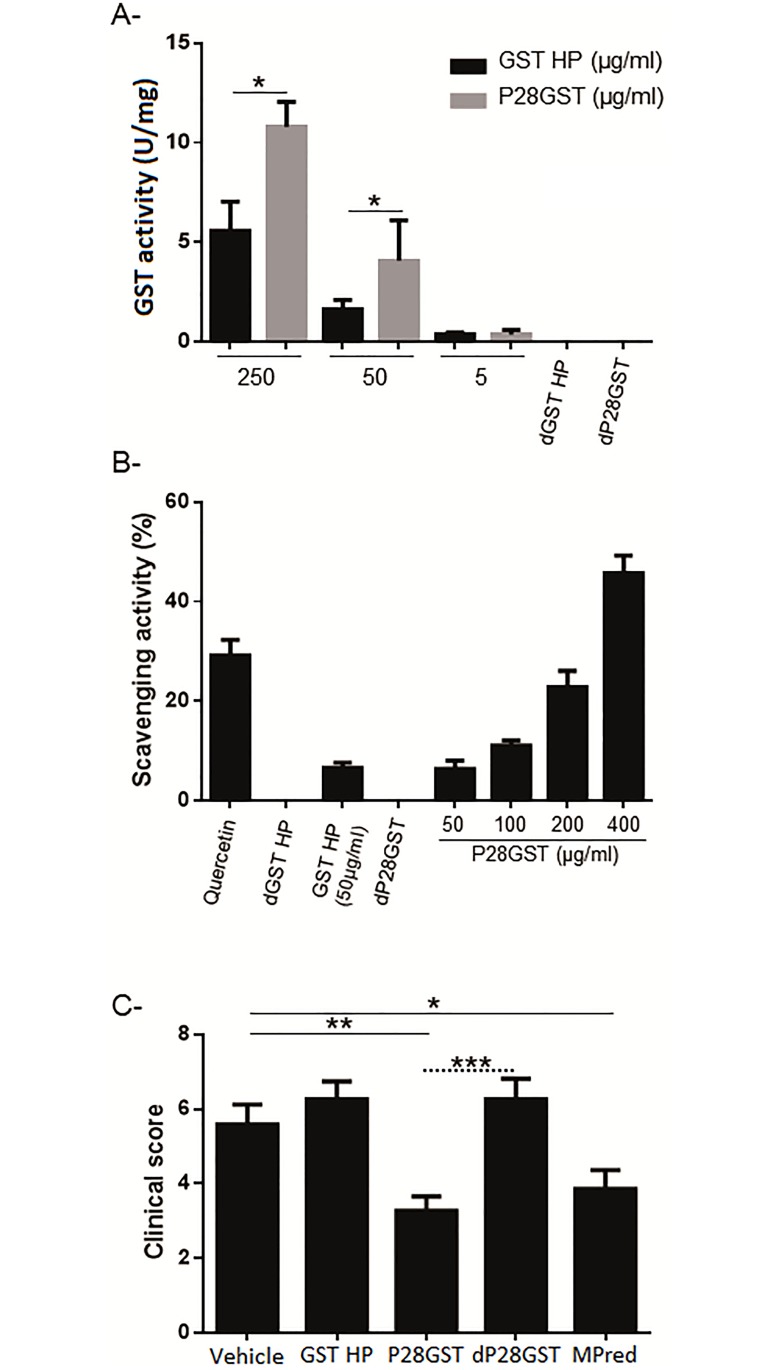
P28 GST enzymatic activity is essential to decrease inflammation in TNBS-induced colitis. A, GST activity was measured in the presence of reduced glutathione at pH 6.5. The GST activity of P28GST is significantly increased when compared to Human Placenta (HP) GST. B, Antioxidant activity of intact or heat-inactivated P28GST or HP GST was determined by ABTS radical scavenging activity. C, Clinical scores obtained after curative treatment of Balb/c mice with TNBS. Balb/c mice were treated with 2 injections of either GST from human placenta (GST HP) and intact or heat-inactivated 5μg/kg P28GST. Data are expressed as means ± SEM from three similar experiments. *p<0.05.; **p<0.01; ***p<0.001.

In order to know whether the GST enzymatic activity of schistosome-derived P28GST was required to exert these anti-inflammatory effects, untreated P28GST was first compared to P28GST denatured by heat-inactivation then to human placenta GST. Fig 4A illustrates the higher GST activity of P28GST when compared to human placenta GST or to heat-inactivated P28GST. In addition, antioxidant activity was measured using the ABTS radical scavenging activity (Fig 4B). The results indicate that P28GST possesses a radical scavenging activity in a concentration dependent manner. Furthermore, the activities obtained with the assay were similar to human placenta GST and quercetin used as positive control at the same concentration. No scavenging activity was detected after heat- inactivation of P28GST and human placenta GST. Finally, as shown in Fig 4C, only intact schistosome-derived P28GST was able to induce a significant reduction of the clinical score, in contrast to heat-inactivated P28GST or human placenta GST.

## Discussion

In the context of the multifactorial nature of IBD, oxidative stress known as a consequence of inflammation has recently moved to potential etiological factor status [21,22]. Indeed, recent data have shown that IBD results in an imbalance between the increase in ROS and a decrease in antioxidant activity explaining many clinical pathophysiological features of CD and UC patients [21–23]. Thus, the degree of severity of the disease as well as the oxidative damage (inflammatory reaction) are correlated with an increase of Nitric Oxide (NO) (production enzyme of the active derivatives of oxygen) [24]. It has also been shown that CD patients have an antioxidant deficiency in their intestinal mucosa. A diet supplemented with antioxidants is consequently recommended [25–27].

Various studies have shown that helminths are able to regulate the immune response of their host to their own benefit. Indeed, via cytokine Th2 production (IL-4, IL-5 and IL-13), they counterbalance the hyper reactive Th1 / Th17 response induced in colitis [28]. In addition, helminths can induce regulatory T cells and alternatively activated macrophages producing IL-10 and TGFβ in order to control the immune response of the host [6]. Indeed, these organisms block effector T cells inducing inflammation by activating dendritic cells and macrophages. They appear to regulate the composition of intestinal flora to their advantage by reducing pro-inflammatory bacteria [29]. All these observations obtained with living parasites have stimulated scientists to develop new tools based on helminths to respond to emerging autoimmune and inflammatory diseases. A research axis has been developed to overcome the various risks associated with the use of worms. This is based on identification, characterization and purification of the parasitic molecules directly involved in the processes of disease improvement. One family of potent immunogenic and pharmacologically active enzymatic proteins, the glutathione-S-transferases (GSTs), has been identified in schistosomes. Among GSTs, a glutathione-S-transferase of *Schistosoma haematobium* (Sh) P28GST has been proposed as the most encouraging anti-parasite and anti-pathology vaccine candidate against schistosomiasis [9,10,30]. This helminth molecule with unique structural feature produced under recombinant form has been characterized from molecular cloning to crystallization [31]. Altogether, our data indicate that P28GST immunization induces a Th2-type response correlated with pathology decrease in several experimental models of schistosomiasis, including non-human primates [9,30]. Phase 1 studies in humans have confirmed the safety and induction of a Th2 response after immunization with Sh28GST [12].

The anti-inflammatory and pro-Th2 properties of P28GST have been recently explored in the prevention of TNBS-induced colitis in rats and mice [13]. We have shown that the treatment based on 3 subcutaneous injections of P28GST in the presence of either aluminum hydroxide [13] or PLGA microparticles [32], prior to colitis induction, reduced intestinal inflammation severity. In addition, these data showed polarization towards a Th2 profile with a decrease in pro-inflammatory Th1 cytokines. Currently, P28GST is being studied in CD patients in a Phase 2 clinical trial (NCT02281916) [7,33]. In order to know the local effect of P28GST, based on its enzymatic activity, we have explored the anti-inflammatory potential of a local injection of P28GST in the absence of adjuvant. Two ip injections of P28GST after administration of TNBS to mice significantly reduced the weight loss as well as the clinical and histological scores. All parameters under study: weight loss and histologic parameters are similarly decreased for the same dose of P28GST. The comparison of P28GST to the reference treatment with Methylprednisolone revealed a similar reduction of clinical and histological scores for the lower dose of P28GST (5μg/kg). As far as the dose is concerned, our results are reproducible for all parameters under study (Figs 1, 2 and 3) indicating that the highest dose of P28GST was not efficient. This was also true in our previous immunization protocol [13] showing that the highest dose of P28GST appeared unable to induce colitis reduction. A possible aggregation, at the highest concentrations, of recombinant P28GST, is under investigation.

As a marker of inflammation in experimental colitis, MPO levels after P28GST curative treatment were evaluated in this study. MPO is a biomarker of neutrophil infiltration characterizing damage of host tissues with inflammation in human and animal model of colitis [34,35]. Local treatment with P28GST after induction of colitis with TNBS at dose 5 and 50μg/kg of P28GST significantly reduced the levels of colonic MPO induced by TNBS in mice. According to the different colon evaluation parameters, the more significant effect was observed at a dose of 5μg/kg. The TNBS-induced colitis model has characteristic features of CD in humans such as severe transmural inflammation, Th1 and Th17 predominant cell-dependent cytokine levels. In fact, pro-inflammatory cytokines (TNF, IL-1β and IL-6) contributed to tissue destruction and colitis perpetuation [36,37]. TNF plays a central role in inflammation to the pathogenesis and one of the actual treatment is based on TNF inhibition. Our results showed that P28GST simultaneously decreased the expression of TNF, IL-1β and IL-6 in local intestinal tissues as well as in peripheral blood. This effect may allow to avoid vicious cycle establishment created by exacerbation of the inflammatory response while allowing these cytokines to continue to perform their physiological function, and probably limit adverse effects due to direct TNF inhibition.

Concerning T cell subsets, data are reinforced by P28GST downregulation on T-bet and RORγt (involved in regulation of Th1/T-bet and Th17/RORγt), unlike Gata-3 and Foxp3 (data not shown). Thus, P28GST treatment induces a significant decrease of Th1/Th17 inflammatory response. Our results suggest that P28GST is able to promote intestinal lymphocytes to counterbalance immune system disorders and decrease the pathology. This modulation by P28GST is accomplished very rapidly, in less than 48 hours, indicating a possible direct mechanism on immune and pro-inflammatory cells. Further studies are needed to explore this possibility.

Our previous results have shown that prevention of colitis by immunization with P28GST in the presence of adjuvant was associated with Th2-type regulatory processes such as eosinophil recruitment in lamina propria or increased IL-13 and IL-5 [13]. The downregulation of Th1 and Th17 cells by P28GST leads to predomination of Th2 cells that can induce M2 macrophages via IL-4 and IL-13 production. The present data indicate that local treatment with P28GST can modify the ratio of Arg (M2) /iNOS (M1) mRNA and thus promote their anti-inflammatory status. These alternatively activated macrophages play a role in the anti-inflammatory response and healing of the tissues. In contrast to M2, conventional macrophages (M1) express inducible nitric oxide synthase (iNOS) and are considered to play a major role in colonic damage in IBD. This is in accordance with previous studies which indicated that resident macrophages are able to modulate their phenotype according to various stimuli present in the inflammatory milieu [28,37,38].

COX enzymes are required for prostanoïds synthesis including prostaglandins, thromboxane, and prostacyclin that exert different action depending on the type of molecule produced and on the specific receptor activated. COX-2 is an inducible enzyme that responds to pro-inflammatory stimuli, whose levels are increased in intestinal inflammation or cancer. PG could promote inflammation of colon epithelial cells by increasing leucocyte and cytokine recruitment [39]. In the present study, COX-2 mRNA levels were significantly reduced by P28GST treatment in the colon tissues in contrast to reference treatment. This suggests that direct or indirect inhibition of COX-2 might be one of the mechanisms by which P28GST can decrease intestinal inflammation. Therefore, P28GST can be considered as a powerful immune-regulator with the ability to strongly decrease the Th1/Th17 response in local and systemic context.

Molecules with antioxidant activity have been reported to alleviate colonic inflammation [40]. We analyzed the antioxidant effect of P28GST by GST activity and free radical scavenging assays. Our results indicate that, as expected, P28GST express a functional GST potential as well as antioxidant activities in vitro. These functions appear fundamental to attenuate the TNBS-induced colitis by reduction of oxidative stress in mouse colon, which is not the case after heat-denaturation of P28GST and HP GST. However, it has to be noticed that the colitis model used in the present work is an acute model, which cannot be totally representative of the chronic inflammatory state observed in IBD. Studies of the effect of P28GST local treatment in a more chronic model (DSS induced colitis) are now in progress. In addition, results of the Phase 2 clinical trial in CD patients, will also give important information concerning the long term effect of P28GST treatment in a chronic inflammatory situation.

## Conclusion

The present results suggest that P28GST which combines immunomodulatory and anti-oxidant properties represents a very promising molecule for the treatment of IBD. First, this compound is a multi-cytokine simultaneous regulator, leading to suppression or at least major decrease of local inflammation. Second, it orchestrates a regulation of the cytokine balance by decreasing the Th1 / Th17 response. Third, its antioxidant effect controls the oxidative damage in colonic tissue. In addition, it already passed with success clinical trials phase 1 for safety and immunogenicity studies. All these arguments indicate that P28GST represents a new and original candidate for the treatment of IBD.
